# Hepatitis B assays in serum, plasma and whole blood on filter paper

**DOI:** 10.1186/1472-6890-12-8

**Published:** 2012-05-20

**Authors:** Theodor K Mayer, Roberto L Vargas, Ann E Knebel, Scott A Williams, Sean P Culver, Daniel M Clark, Louise R King

**Affiliations:** 1 Department of Pathology and Laboratory Medicine, Rochester General Hospital, 1425 Portland Ave, Rochester, NY, 14621, USA; 2 Department of Chemistry, Rochester Institute of Technology, 85 Lomb Memorial Drive, Rochester, NY, 14623, USA; 3 Printing Materials and Applications Laboratory, Rochester Institute of Technology, Rochester, NY, 14623, USA; 4 Shyira Hospital, B.P. 26, Ruhengeri, Shyira, Rwanda

**Keywords:** Hepatitis B, Dried blood spot, Surface antigen, Surface antibody

## Abstract

**Background:**

Screening and determining the immune status of individuals for hepatitis B is usually done by detecting hepatitis B surface antigen (HBsAg) and hepatitis B surface antigen-specific antibodies (HBsAb). In some countries with the highest viral burden, performing these assays is currently impractical. This paper explores the use of filter paper as a blood specimen transport medium.

**Methods:**

Samples, chosen from routine clinical laboratory pool, were applied and dried onto filter paper. Eluates, from the paper samples, were analyzed as routine clinical specimens on ADVIA Centaur 5634® immunoassay analyzers using the standard HBsAg and HBsAb kits. Dried blood samples were subjected to a range of environmental conditions in order to assess stability.

**Results:**

After drying and elution the assays showed linearity and precision comparable to clinical assays performed on fresh serum. Elutions at various times during a 149 day incubation period showed very little variability in the Index numbers. All analytes were temperature stable except for a decrease in the HBsAg signal at 42°C.

**Conclusions:**

Filter paper is an acceptable storage and transport medium for serum to be used in the detection of hepatitis B markers if atmospheric variability can be controlled. HBsAg, HBsAb and HBcAb are all stable for at least five months under storage conditions below room temperature. Drying specimens, particularly serum, on filter paper at remote locations, offers a reasonable solution to the problem of hepatitis surveillance in underdeveloped regions, although some attempt at temperature control might be desirable.

## Background

Hepatitis B virus is a human pathogen that infects the liver and can cause both acute and chronic disease. More than 350 million individuals live with chronic hepatitis B worldwide [1,2]. These individuals are often asymptomatic but approximately 25% of adults who are chronically infected will die of cirrhosis or hepatocellular carcinoma secondary to the infection [3]. The best approach to reduce the burden of hepatitis B is to prevent infection, mainly through vaccination and infection control measures [4]. There are also treatment options with some effectiveness that include interferon, anti-viral drugs and in some cases liver transplants.

Screening for disease and determining the immune status of individuals is usually done by detecting hepatitis B surface antigen (HBsAg), hepatitis B surface antigen-specific antibodies (HBsAb) and hepatitis B core antibodies (HBc). The presence of anti-HBsAg IgG is a marker for immunity and used to determine whether a patient needs to be vaccinated [5,6]. The most commonly used tests are immunoassays performed on fairly complex analyzers by trained technologists [5-7]. In some countries with the highest viral burden, such as those in sub-Saharan Africa, performing these assays is currently impractical due to the local unavailability of laboratory resources.

Blood serum and plasma specimens have in many instances been successfully collected and dried onto paper media for subsequent testing elsewhere [8-17]. This paper explores the use of filter paper as a medium on which to apply and dry serum or plasma specimens in the field for safe and convenient transport to laboratories possessing the required technology for the testing of hepatitis B markers.

## Methods

### Specimen collection and immobilization onto paper

Specimens were chosen from the routine clinical laboratory at the Rochester General Hospital for study, representing the different laboratory presentations seen in patients, including immunization, current chronic or acute disease, and resolved infection. The specimen collection plan was submitted and found to be exempt from formal review by the Internal Review Board (IRB) at the Rochester General Hospital. The Institutional Review Board deemed this study to be exempt under Federal regulations after all patient identifiers were stripped from the samples by a third party not involved in the research before being tested. 50 μl aliquots of the specimens were absorbed into fifteen centimeter diameter filter paper (Reeve Angel® 230- Whatman Inc.) and allowed to dry by hanging at ambient temperature for a day. This paper has a high absorptive capacity (5.0 ml per filter) for serum and plasma (data not shown). Dried paper was stored individually in plastic bags at room temperature (21°C). No silica gel desiccants were used in the plastic bags.

### Disc elution

Paper discs were obtained from the filter paper by using a hand punch (M.C. Mieth Manufacturing Inc.). Each paper disc measured 8.73 mm. The specimens were eluted with deionized water and pH 7.2 phosphate buffered saline (Beckman-Coulter®) in watch glasses, using the same volume of sample that had been originally applied to the filter paper. Some discs required tamping to insure complete elution. The discs were stacked in threes in the watch glasses and placed into the cylinders of three milliliter disposable syringes (BD 3ml syringe – Luer-Lok™ Tip) using metal forceps. The stacks were tapped to the bottom of the syringes using a blunt rod, and seated flat. The discs were eluted under gravity into 16 × 100 mm tube (Vacutainer) with no additives. When no liquid was visible above the filter discs, final elutions were made using the syringe plungers. The punch, watch glass, forceps and blunt rod were thoroughly rinsed and dried between specimens.

### Hepatitis B analysis and characterization

Eluates were analyzed as routine clinical specimens on ADVIA Centaur 5634® immunoassay analyzers (Siemens) for surface antigen (HBsAg), total core antibody (HBcAb), and surface antibody (HBsAb) following the manufacturer recommendations. The ADVIA Centaur assays are magnetic particle chemiluminometric immunoassays that use biotinylated mouse monoclonal capture antibodies and acridinium-ester labeled mouse monoclonal antibodies and streptavidin coated magnetic latex particles. The cutoff Index value for positivity is ≥1.00 including results between 1.00-1.95 Index value which are considered weakly reactive [18].

To characterize the properties of the samples after dehydration and elution, we performed studies of linearity, precision, and stability at different conditions. The linearity studies were conducted using serum from two different patients, one for antigen, and the second for the two antibodies. The high HBsAg titer sample (26,000 relative light units in the original assay) was serially diluted from 1:5 to 1:10,000. HBcoreTotal and HBsAB linearity studies were performed on dilution from 1:2.5 to 1:10,000. The precision studies were performed by making 136 punches from a saturated 15.0 cm filter. These discs were mixed in the same plastic bag in which the filter was stored. Five groups of fifteen random discs were removed, and the specimens were eluted and assayed.

 The correlation of results was described using R-squared analysis, linear regression and a simple degree of concordance calculation. Coefficient of variation was defined as the ratio of the standard deviation to the mean.

### Specimen source, stability and uniformity

The first stability experiments were carried out on paper saturated with a pool containing all three markers made from different individual specimens. Samples were kept at room temperature and assayed at one-week intervals for the first month then monthly for four months, for the three hepatitis B markers.

To determine the stability of the three markers over time at different temperatures, a single 15 cm filter saturated with the pool was used to obtain seventy-two filter paper discs. The discs were mixed in a plastic bag. Three discs were placed into each of 24 glassine envelopes (Westvāco – Envelope Division). The envelopes were stored from one day to 28 days under following conditions: ambient temperature (77.5°F), refrigerator temperature (4°C), freezer (−18°C), deep freeze (−78°C), and 42°C incubator. An additional set of three discs was exposed to gamma radiation from a cesium-source blood irradiator for seven minutes and 12 seconds. The effect of humidity was studied by placing five 15.0 cm filter papers into environmental chambers at each of the following humidity levels: 20%, 50% and 90%.

To determine whether significant differences in analyte recovery occurred regionally across the filter, a 15.0cm filter was divided into 8 sectors, and each sector was subdivided into a central and a peripheral section. Including a specimen from the actual center of the filter, a total of 17 regions were assayed from the same original filter paper.

While the assays are preferentially performed on serum specimens, we attempted to determine the suitability of plasma and whole blood as well. Serum, plasma, and ethylenediaminetetraacetic acid (EDTA) whole blood specimens were obtained via venipuncture from positive patients and negative volunteers. Both plasma and serum specimens were analyzed for baseline levels of the three analytes. Serum, plasma, and whole blood were applied to individual filters and dried. The papers were processed as described. Tandem samples from three patients were processed to compare serum and plasma.

## Results

### Assay linearity

**Figure 1:**
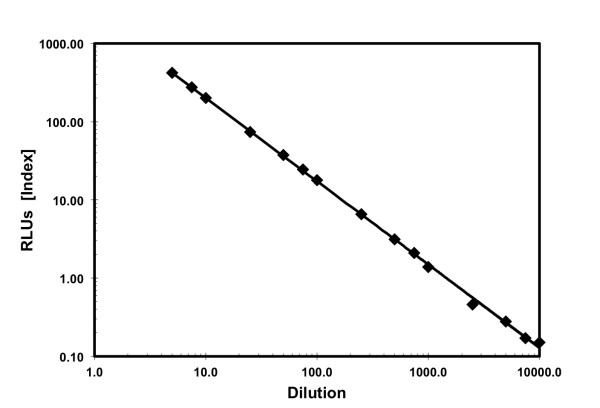
**HBsAg Linearity and Dynamic Range Testing Following Paper Elution.** The measured versus calculated results exhibited a correlation coefficient (r^2^) of 0.9992, over a dynamic range of 0.10 to 1000 RLUs (Index), when compared to the results obtained from the fresh specimens. The RLU-Index values are provided for comparison.

After drying and elution the assays showed linearity and precision comparable to clinical assays performed on fresh serum. HBsAg testing after elution had a dynamic range of 0.10 to 1000 Relative Light Units (RLUs; Index) with a correlation coefficient R^2^ equal to 0.9992 when compared to the results obtained from the fresh specimens. The results for HBcAb and HBsAb were linear from 1:2.5 to 1:100 and well correlated with the previously determined results. The dilutions from 1:250 to 1:10,000 were also statistically well correlated (Figure 1). The coefficient of variation of the assays was less than 10%. The HBsAg range was 17127–19494. The HBcAb range (Index) was 105.51–114.14. The HBsAB range (Index) was 47.24–54.19.

### Specimen stability

**Figure 2:**
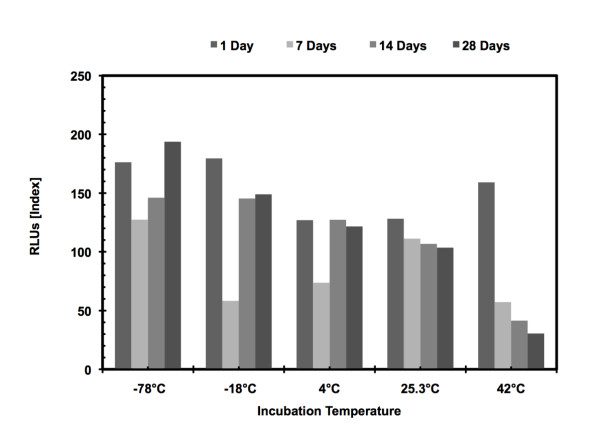
**Temperature Stability of the HBsAg Assay.** Comparison of signal strength from samples stored at −78°C, -18°C, 4°C, 25.3°C and 42°C for up to 28 days. Samples stored at all tempreatures were stable except for the samples stored at 42°C which degraded over time.

Elutions at various times during the 149 days showed very little variability in the Index numbers produced by the assays with all three analytes being stable at room temperature and below (−70 to 25.3°C). However, after exposure to high atmospheric temperature (42°C) for an undetermined amount of time signal was lost and the targets denatured to the point that they could not be detected by the assay. Representative data for HBsAg is detailed in Figure 2. The HBsAg range was 12151–15820 RLUs. The HBcAb range was 105.68–140.90 (RLUs). The HBsAb range was 72.04–92.09 (RLUs). The calculated coefficients of variation were less than 10%.

The HBsAb values were stable at all temperatures studied. The HBcAb value was stable at all lower temperatures, but decreased 25% at 7 days and 33% at 14 days at 42°C. The HBsAg concentration was stable at all lower temperatures, but showed decreases of 55% at 7 days and 68% at 14 days at 42°C.

### Specimen source

The whole blood elution showed a significant increase in HBsAb, while the HbsAg and HBcoreTotal results were unaffected by the presence of hemolysis. There was no significant difference in the data obtained from the three humidity levels of filter paper for HBsAg, HBsAb and HBCore Total. Identical negative results were obtained from serum and plasma of patients with no hepatitis B markers.

A mapping study (data not shown) showed little variation in HBsAg results across all sectors tested. The range of variability went from 17% above and 13% below the average signal value in all regions that is within acceptable performance parameters. This variability did not affect the interpretation of results for HBsAg.

**Figure 3:**
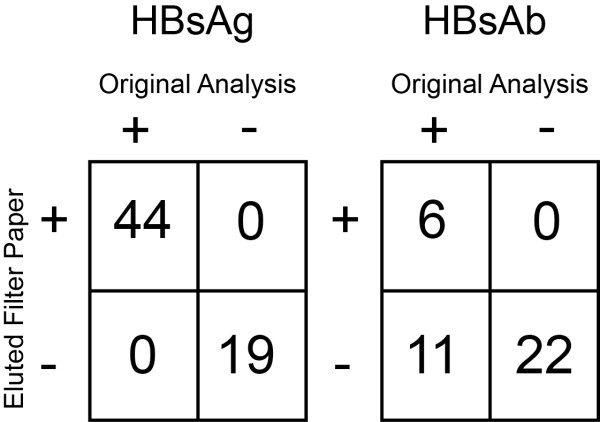
**Comparison of the (a) HBsAg (n = 63 samples) and (b) HBsAb (n = 39 samples) interpretation (positive, negative or equivocal) between initial patient clinical results (serum specimens) and subsequent results obtained from eluted filter papers.** The eleven discrepant results in the HBsAb assay were related to very low signal in the original samples.

Overall, there was exact agreement in the HBsAg interpretation (positive, negative or equivocal) between initial patient clinical results (serum specimens) and subsequent results obtained from eluted filter papers (Figure 3 a,b). Many of the false negatives had original serum values below 10 which is considered not immune.

## Discussion

Filter paper is an acceptable storage and transport medium for serum to be used in the detection of hepatitis B markers if temperature can be controlled. HBsAg, HBsAb and HBcAb are all stable for at least five months under storage conditions at or below 25^0^C. The test characteristics of linearity and precision were unchanged when compared to the assays performed on fresh serum. Humidity had no effect on the measurements obtained. At 42°C there was a significant decrease in the measured HBsAg. While the decrease was marked, interpretation of the Index value did not change and the result would have still been reported as positive for HBsAg. There was also a slightly increased coefficient of variation at 42°C although the difference would not affect the final interpretation of the values. We did not find a single discrepant result when compared on the basis of interpretation (positive or negative for markers). Serum is the preferred testing sample rather than whole blood since there is some positive bias for surface antibody when using whole blood. This interference may be caused by hemoglobin on the filter paper. Plasma did not show any background interference.

Clearly, DBS techniques have been investigated for decades as a means of extending the reach of clinical services into remote regions. Our results build on recent prior studies that, collectively suggest, whole blood may be collected and stabilized on a variety of different filter papers [14-16,19,20]. Our results also confirm that the HBsAg, reconstituted from immobilized dried whole blood, was most sensitive to changes in ambient temperature [20,21]. Our results, however, do provide a pathway to DBS collection validation when coupled with the sensitive chemiluminescent immunoassay technique. In our study, all three markers remained above the positive cutoff even when stored at 42°C for 28 days. This period would be long enough to survive mailing in tropical or remote regions. To our knowledge, our study is the first to suggest that all three markers for hepatitis B may be immobilized and dried onto filter paper, transported at elevated temperatures over an extended period of time without refrigeration, and reconstituted to provide a positive assay using a sensitive immunochemical analysis.

## Conclusions

Drying specimens, particularly serum, on filter paper at remote locations, offers a reasonable solution to the problem of hepatitis surveillance in underdeveloped regions, although some attempt at temperature control might be desirable. Analytes are stable, and the papers can be shipped to centralized facilities that have advanced testing capabilities. Our proof-of-concept work, using an immunofluorescence methodology remains to be validated in field trials. These field trials are currently under way in Rwanda.

## Abbreviations

DBS, Dried blood spot; HBsAg, Hepatitis B surface antigen; HBsAb, Hepatitis B surface antibody; HBcAb, Hepatitis B core antibody.

## Competing interests

The authors declare that they have no competing interests.

## Authors’ contributions

TKM, RLV, LRK and SAW conceived of the study, and participated in its design and coordination and helped to draft the manuscript. AEK and SPC designed and carried out the filter paper immobilization and reconstitution procedures. DMC designed and carried out the environmental studies related the impact of temperature on the assay. DMC also designed and carried out the measurements on the physical properties of the filter paper media. All authors read and approved the final manuscript.

## Author information

TKM is the Chief of Laboratory Services and RLV is the Director of Microbiology in the Department of Pathology and Laboratory at the Rochester General Hospital. LRK is a physician of internal medicine and currently practices at the Shyira Hospital in Rwanda, Africa which serves a surrounding rural population of over 200,000. SAW is a Professor of Chemistry at RIT, and with DMC, designed and coordinated research involving the material properties related to the paper and biomaterials used in the study. Our multi-disciplinary team is part of the RGH-RIT collaboration between local hospitals and academic departments.

## Pre-publication history

The pre-publication history for this paper can be accessed here:

http://www.biomedcentral.com/1472-6890/12/8/prepub
